# Insight into the mechanism of action of ORG27569 at the cannabinoid type one receptor utilising a unified mathematical model

**DOI:** 10.1007/s00210-023-02923-6

**Published:** 2024-01-16

**Authors:** Hayley M. Green, Liang Yang, Xiao Zhu, David B. Finlay, Stephen B. Duffull, Michelle Glass

**Affiliations:** 1 Department of Pharmacology and Toxicology, School of Biomedical Sciences, University of Otago, PO Box 56, Dunedin, 9054 New Zealand; 2 Otago Pharmacometrics Group, School of Pharmacy, University of Otago, Dunedin, New Zealand; 3 Department of Clinical Pharmacy and Pharmacy Administration, School of Pharmacy, Fudan University, Shanghai, China; 4 Certara, Princeton, NJ USA

**Keywords:** Cannabinoid type 1 receptor, ORG27569, Cyclic adenosine monophosphate (cAMP)

## Abstract

Allosteric modulation of CB_1_ is therapeutically advantageous compared to orthosteric activation as it potentially offers reduced on-target adverse effects. ORG27569 is an allosteric modulator that increases orthosteric agonist binding to CB_1_ but decreases functional signalling. ORG27569 is characterised by a delay in disinhibition of agonist-induced cAMP inhibition (lag); however, the mechanism behind this kinetic lag is yet to be identified. We aimed to utilise a mathematical model to predict data and design in vitro experiments to elucidate mechanisms behind the unique signalling profile of ORG27569. The established kinetic ternary complex model includes the existence of a transitional state of CB_1_ bound to ORG27569 and CP55940 and was used to simulate kinetic cAMP data using NONMEM 7.4 and Matlab R2020b. These data were compared with empirical cAMP BRET data in HEK293 cells stably expressing hCB_1_. The pharmacometric model suggested that the kinetic lag in cAMP disinhibition by ORG27569 is caused by signal amplification in the cAMP assay and can be reduced by decreasing receptor number. This was confirmed experimentally, as reducing receptor number through agonist-induced internalisation resulted in a decreased kinetic lag by ORG27569. ORG27569 was found to have a similar interaction with CP55940 and the high efficacy agonist WIN55,212-2, and was suggested to have lower affinity for CB_1_ bound by the partial agonist THC compared to CP55940. Allosteric modulators have unique signalling profiles that are often difficult to interrogate exclusively in vitro. We have used a combined mathematical and in vitro approach to prove that ORG27569 causes a delay in disinhibition of agonist-induced cAMP inhibition due to large receptor reserve in this pathway. We also used the pharmacometric model to investigate the common phenomenon of probe dependence, to propose that ORG27569 binds with higher affinity to CB_1_ bound by high efficacy orthosteric agonists.

**Supplementary Information:**

The online version contains supplementary material available at 10.1007/s00210-023-02923-6.

## Introduction

The cannabinoid type-1 receptor (CB_1_) is a G protein-coupled receptor (GPCR) found predominately in the central nervous system (Pertwee and Ross 2002). CB_1_ is expressed presynaptically and is activated via retrograde neurotransmission, in which endocannabinoids (AEA and 2-AG) are released from the postsynaptic neuron, traverse the synapse, and bind to the orthosteric site on CB_1_, ultimately inhibiting the release of other neurotransmitters (Katona et al. 2008). CB_1_ has been identified as a suitable drug target for multiple sclerosis, Huntington’s disease, epilepsy, pain-related disorders, obesity, and other neurological diseases, but its therapeutic potential is limited by on-target adverse effects (Haspula and Clark 2020). Allosteric modulators are compounds that bind to sites topographically distinct from the orthosteric site and are suggested to possess safer therapeutic profiles than orthosteric exogenous drugs by retaining the spatial and temporal tone of endogenous ligands (Wootten et al. 2013). Allosteric compounds act by causing conformational changes in the receptor that influence the orthosteric binding site and/or effector coupling (Christopoulos and Kenakin 2002).

The first allosteric modulators of CB_1_ were developed by Organon Research, ORG27759, ORG29647, and ORG27569 (here-on, “ORG”)—the most potent compound (Price et al. 2005). Allosteric compounds bind to a site topographically distinct from the orthosteric binding site (Shao et al. 2019). At CB_1_, uniquely, ORG, and similar compounds increase the binding affinity of orthosteric agonists but decrease G protein signalling (Kenakin and Strachan 2018). In accordance with the operational model of allosteric modulation, this correlates to positive binding cooperativity (α) and negative functional cooperativity (β). Real-time analysis of ORG on agonist-mediated inhibition of cyclic adenosine monophosphate (cAMP) identified that ORG caused time-dependent modulation of agonist signalling (Cawston et al. 2013). This manifested in a kinetic lag in which agonist-mediated inhibition of cAMP appeared normal for several minutes, after which the agonism was attenuated. This was suggested to occur due to an increased rate of receptor desensitisation and reduced receptor internalisation (Cawston et al. 2013).

Another proposed advantage of allosteric modulators is probe dependence, more specifically their ability to result in varying functional outcomes in combination with different orthosteric agonists (Wootten et al. 2013). ORG has been shown to have lower potency at inhibiting WIN55,212-induced cAMP inhibition compared to in combination with CP55940 (Baillie et al. 2013). More recently ORG has been shown to stabilise a different conformation of CB_1_ in combination with the endocannabinoid 2-AG, compared with other orthosteric agonists, suggesting probe dependence (Wang et al. 2021).

**Fig. 1:**
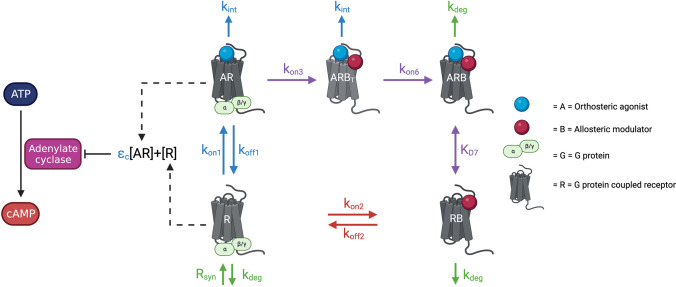
Schematic of the reduced model describing the receptor species involved in the binding of CP55940 and ORG to CB_1_ and their involvement in cAMP inhibition (published in Yang et al. 2023). The agonist bound receptor (AR) and constitutively active receptor (R) both contribute to the inhibition of cAMP production. The orthosteric agonist (A, CP55940) is expressed by a blue sphere, and all parameter values pertaining to the orthosteric agonist are also expressed in blue. The allosteric modulator (B, ORG27569) and the parameter values associated with the allosteric modulator are shown in red. The relationship between the transitional state receptor (ARB_T_), inactive receptor bound by both orthosteric and allosteric ligands (ARB), and the allosteric bound receptor (RB) is shown, and parameters that pertain to the interaction between orthosteric and allosteric ligands are shown in purple. System parameters are shown in green. A full list of parameters and their definitions are described by Yang et al. (2023). Figure created in Biorender.com

Recently, we published a unified mathematical model describing the effect of ORG on the signalling of the CB_1_ orthosteric agonist CP55940 (Fig. 1; Yang et al. 2023). This model describes previous in vitro data and comprises a two-part model explaining receptor binding and internalisation and downstream cAMP signalling (Cawston et al. 2013; Yang et al. 2023). Inhibition of cAMP is a complex process involving G proteins, adenylate cyclase, and other modulators and molecules, but in this model, constitutively active receptor (*R*) and agonist bound receptor (*AR*) directly inhibit cAMP (Yang et al. 2023). This was done to simplify the model; however, the model could be expanded in the future to include signalling molecules and more accurately represent the biological system. Within the model, *A* fers to CP55940, *B* refers to ORG, and *R* refers to CB_1_ (Fig. 1; Yang et al. 2023). This study concluded that the existence of a transitional state (*ARB*_*T*_) and an inactive state *ARB*, when both ORG and CP are bound to CB_1_, are imperative to describe all signalling data (Fig. 1; Yang et al. 2023). This is a unique feature of this model, as classical allosteric ternary complex models do not have the existence of such a transitional state. The transitional state proposed cannot signal through G proteins but is able to undergo internalisation (Yang et al. 2023). Importantly, this model proposes an empirically testable mechanism of action for ORG at CB_1_.

An intermediate activity state has been proposed in several studies to help explain the effects of ORG at CB_1_, and this has been elaborated upon in both functional, structural, and theoretical research, although the exact nature of these proposed states differs slightly (Fay and Farrens 2012, 2015; Cawston et al. 2013; Shao et al. 2019; Wang et al. 2021). The unique time-dependent attenuation of cAMP inhibition by ORG was hypothesised to arise from the stabilisation of an inactive receptor conformation that differs from the conformation stabilised by the inverse agonist SR141716A (Cawston et al. 2013). This conformation was suggested to cause a partial blockade of constitutive activity, therefore resulting in significantly lower cAMP production than was observed in response to SR141716A (Cawston et al. 2013). In the recent CB_1_ crystal structure study, ORG was found to compete with endogenous cholesterol for a cholesterol binding site and stabilise an intermediate state of CB_1_ (Shao et al. 2019). This intermediate state allowed an orthosteric agonist to bind (therefore resembling an active state conformation extracellularly) but did not allow G protein coupling due to restricted movement of transmembrane helix (TMH) 6 (Shao et al. 2019). Studies utilising site-directed fluorescence labelling have also suggested that ORG stabilises an intermediate structure on the pathway to receptor activation (Fay and Farrens 2012, 2015). When the inverse agonist/antagonist SR141716A interacts with CB_1_, movement of TMH6 is restricted (Fay and Farrens 2015). SR141716A also restricts movement at helix 8/TMH7, whereas this study suggested that ORG enhances conformational changes in helix 8/TMH7, a similar movement to that observed by agonists at CB_1_ (Fay and Farrens 2015). It has been suggested that β-arrestin biased ligands favour receptor conformational changes around TMH7, and G protein biased ligands favour TMH6 movements (Liu et al. 2012; Rahmeh et al. 2012). This suggestion aligns with the characteristics of the transitional state (ARB_T_) proposed in the mathematical model and validated in this study, such that ORG stabilises a conformation of hCB_1_ that can internalise and therefore recruit β-arrestin proteins (Yang et al. 2023). The exact definitions of these unique conformational states vary slightly to the transitional state proposed in Yang et al. (2023). However, the structural data detailing the inability of an intermediate state to allow G protein binding aligns with the functional description of the transitional state we propose (Fay and Farrens 2012, 2015; Cawston et al. 2013; Shao et al. 2019; Yang et al. 2023).

This study builds on experimental findings first observed in Cawston et al. (2013) and aimed to use the established mathematical model (Yang et al. 2023) to predict data and design in vitro experiments to gain mechanistic insight into the unique signalling profile of ORG. As described by Yang et al. (2023), the model was reduced by removing parameters estimated close to 0, assuming quasi-equilibrium for *ARB* (agonist-receptor-allosteric ligand complex) and *RB* (receptor-allosteric ligand complex), and lumping *RB*_*T*_ (transitional state of receptor-allosteric ligand complex) with RB (Yang et al. 2023). In this study, we used parameter values estimated in Yang et al. (2023) to simulate experimental data prior to experimentation. As the established model was built on existing data, we first validate the ability of the model to predict new data using a simple in vitro experimental design that extends the range of concentrations of the agonist utilised in original experiments. Following this, the model was used to design experiments to further understand the mechanism behind the kinetic lag in cAMP disinhibition by ORG. As probe dependence has been suggested in vitro, we explore the ability of the model to be extended to predict for different orthosteric ligands in combination with ORG.

## Materials and methods

### Drugs

*Rel*-5-(1,1-dimethylheptyl)-2-[(1R,2R,5R)-5-hydroxy-2-(3-hydroxypropyl)cyclohexyl]-phenol (CP55940) was purchased from Cayman Chemical Company (Ann Arbor, MI, USA) and stored at 10 mM in absolute ethanol. 5-Chloro-3-ethyl-N-[2-[4-(1-piperidinyl)phenyl]ethyl-1H-indole-2-carboxamide (ORG27569) was purchased from Selleck Chemicals (Houston, TX, USA) and stored at 31.6 mM in DMSO. All drugs were aliquoted into single-use aliquots and stored at −80 °C. Forskolin was also purchased from Cayman Chemical Company and stored at 31.6 mM in DMSO in large, multiuse aliquots.

### Cell lines and maintenance

Human embryonic kidney 293 (HEK293) cells stably expressing triple-hemagglutinin-tagged human CB_1_ (Cawston et al. 2013) were used for all assays. HEK293/3HA-hCB_1_ cells were cultured in high glucose Dulbecco’s Modified Eagle Medium (DMEM) containing 10 % fetal bovine serum (FBS) and supplemented with 250 μg/ml zeocin, as previously described (Cawston et al. 2013). Cells were maintained in a 75 cm^2^ vented-cap flask in a humidified 37 °C incubator with 5% CO_2_.

### General cAMP CAMYEL assay method

Cellular cAMP was measured with a real-time bioluminescence resonance energy transfer (BRET) assay, utilising the CAMYEL biosensor (ATCC MBA-277; Jiang et al. 2007; Cawston et al. 2013).

Assays were completed as previously described by Cawston et al. (2013). Briefly, HEK293/3HA-hCB_1_ cells were seeded at a density of 5 × 10^6^ into 10 cm culture dishes (Corning, Corning, NY) and incubated at 37 °C for 24 h to achieve 50–60% confluence. Medium was replaced and cells were transiently transfected using linear polyethylenimine (PEI, Polysciences, Warrington, PA) at a 1:6 DNA:PEI ratio (5 μg pcDNA3.1-His-CAMYEL:30 µg PEI). Transfection mixture was prepared in 150 mM sodium chloride and dispensed dropwise into culture dishes, and cells were incubated at 37 °C for 24 h. Transfected cells were then trypsinised and replated into poly-d-lysine (PDL, Sigma-Aldrich) coated, white 96-well CulturPlates (PerkinElmer) at a density of 50,000 cells/well and incubated for a further 24 h. To assay, cells were washed with PBS and medium was replaced with BRET assay medium (phenol red-free DMEM containing 25 mM HEPES and 1 mg/ml fatty acid free bovine serum albumin: BSA, MP Biomedicals, Auckland, NZ). Drugs were prepared in BRET assay medium at required concentration in the presence of 5 µM forskolin and combined in a polypropylene V-well plate. Coelenterazine-h (final concentration of 5 µM; Nanolight Technology, Prolume Ltd) was dispensed to cells, and luminescence (475 nm and 535 nm) was detected simultaneously in real-time using a LUMIstar Omega plate reader for 5 min, or cells were incubated in the dark at 37 °C for 5 min prior to drug addition. Drugs were then added to cells using a multichannel pipette (final volume 100 µL), and luminescence (475 nm and 535 nm) was detected simultaneously in real-time using a LUMIstar for the appropriate duration of time (see results). BRET data were exported as inverse BRET ratios (475/535 nm) from Omega MARS software (V3.1 R5, BMG Labtech GmbH) into Excel, and all subsequent analyses were performed using GraphPad Prism v8 (GraphPad Software Inc., La Jolla, CA, USA).

### Validation of the mathematical model

The experimental cAMP data used in establishing the mathematical model included one concentration of CP55940 (1 µM; Cawston et al. 2013; Yang et al. 2023). To validate the model with external data, new cAMP assays included multiple concentrations of CP (0 to 10 µM).

These new data were overlayed by model predictions with 95% confidence intervals (CI) from a global sensitivity analysis. The 95% CI was based on the full variance-covariance matrices of parameter estimates by NONMEM 7.4 from the mathematical model (Yang et al. 2023). The distribution of the parameter estimates is multivariate normal, with the variance-covariance matrix that can be estimated from the data according to asymptotic statistical theory (Jonsson and Bauer 2012). The full variance-covariance matrices of parameters were recorded in the ‘.cov’ output file generated by NONMEM during estimation in the mathematical model.

Baseline and forskolin efficacy of each experiment were adjusted according to experimental results. The residual error was added to the simulations (as per Yang et al., (2023)). The model was considered to be validated if the observations were consistent with the model predictions, i.e. where the observations fell within confidence intervals of predictions.

### Model-assisted mechanism exploring the kinetic lag in cAMP disinhibition by ORG

The allosteric model (Yang et al. 2023) was used to simulate kinetic cAMP inhibition results and the influence of each possible receptor species and their interactions on total cAMP inhibition. The species of the allosteric model include CB_1_, CP-CB_1_, CB_1_-ORG, and CP-CB_1_-ORG in both transitional and inactive states. From this, the species that contribute significantly to the kinetic lag in cAMP disinhibition can be identified. The simulations and calculations were done by MATLAB R2020b.

cAMP experiments were then designed to test the model-proposed hypotheses where the experiment was performed in alignment with the general cAMP CAMYEL assay method described above. The hypothesis was verified if the model-predicted delay time *T*_d_ of allosteric modulation onset and experimentally observed *T*_d_ were comparable. The definitions of model-predicted delay time *T*_d_ and experimentally observed *T*_d_ are introduced below.

#### Definitions of the kinetic lag in cAMP disinhibition by ORG 

##### (1) Definition of the model-predicted kinetic lag

**Fig. 2:**
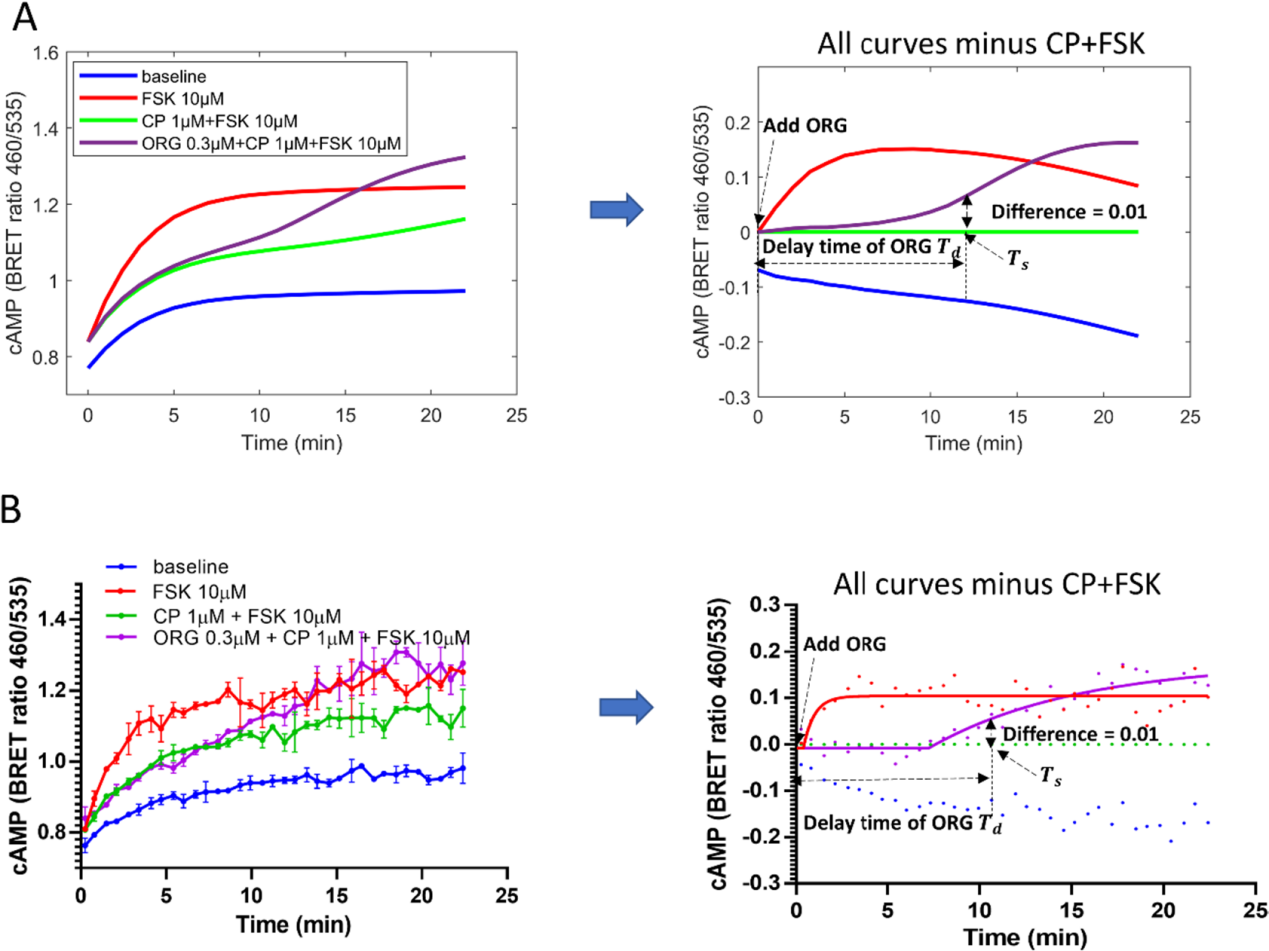
Definitions of the kinetic lag in cAMP disinhibition by ORG. **A** The curves of different conditions were simulated by the allosteric model. The right panel was generated from the left panel by subtracting the CP+FSK curve. Separation of the curves from the curve CP+FSK was determined when the difference between the curve ORG+CP+FSK and the curve CP+FSK (*y* = 0 line in right panel) exceeded a set level of tolerance (set to 0.01 in these simulations), and the time of separation (T_S_) is recorded. The time from the addition of ORG in the experiment to the separation time (T_S_) is defined as the lag of ORG (T_d_). **B** The experimental data arise from different concentrations of ORG and CP, data from Cawston et al. (2013). The right panel was generated from the left panel subtracting the CP+FSK curve. The curves in the right panel were then fit with the equation of one-phase association with plateau*.* The curve fits are shown as smooth lines and data are shown as points. The time of separation (T_S_) was defined as the minimum time for the difference between the fitted curves ORG+CP+FSK and the curve CP+FSK (*y* = 0 line in the right panel) to exceed 0.01. The time after adding ORG and before the separation is the ORG lag (T_d_)

Within the model, the lag in disinhibition of cAMP by ORG is defined as the time prior to the divergence of CP55940 conditions containing ORG from CP55940 alone. First, the model subtracts the predicted curve of CP55940 alone (in the presence of forskolin), from all predicted curves containing ORG. Separation of the curves was determined when the difference between the curves exceeded some level of tolerance (set to 0.01 in these simulations), and the time of separation (*T*_S_) is recorded. The time from the addition of ORG in the experiment to the separation time (*T*_S_) is termed the lag of ORG (*T*_d_) (Fig. 2A). Note, when ORG was added at time=0 then *T*_d_=*T*_s_. If ORG is not added at time=0, then *T*_d_ equals *T*_s_ minus administration time of ORG.

Simulation of data to calculate the delay time *T*_d_ was performed without random effects, therefore representing a population average delay time.

##### (2) Definition of the experiment-observed kinetic lag

As empirical data contains experimental error, a different method is used to calculate the lag. Using GraphPad Prism v8, the mean of CP55940 in the presence of forskolin is subtracted from all conditions, normalising the data where CP55940 alone is equal to zero. Data is then fit with the ‘plateau followed by one phase association’ equation (Cawston et al. 2013). When the difference of the curves in the presence and absence of ORG (CP+FSK+ORG and CP+FSK, respectively) is 0.01, the curves were deemed to be sufficiently separated. The time between the addition of ORG and where the curves separate is defined as *T*_*d*_ (Fig. 2B). This separation time can be calculated using the interpolation function of PRISM.

### Exploration of orthosteric-dependent ORG effects

The model by Yang et al. (2023) was expanded to include the orthosteric ligands WIN55,212-2 and THC. Within the model, there are three orthosteric ligand-specific parameters that are built off data pertaining to CP55940 (Yang et al. 2023). These include the conventional ternary complex model parameters to describe binding and internalisation of CP55940 at CB_1_, in combination with the operational model parameters that describe the functionality of CP55940 in cAMP inhibition (Yang et al. 2023). The binding affinity of CP55940 is defined by the equilibrium dissociation constant, Kd; the efficacy of CP55940 is described by the parameter *eps* (Zhu et al. 2019); and the internalisation rate constant *k*_*int*_ defines the rate at which CP55940 causes internalisation of CB_1_. As these three parameters pertain only to the orthosteric agonist (ligand A) in the model, to allow the model to make predictions for orthosteric agonists other than CP55940, these orthosteric ligand-specific parameters were changed to capture the interaction each orthosteric agonist has with CB_1_ in the absence of ORG. All parameters in the model that describe ORG (ligand B) or the interaction between orthosteric ligand and ORG were kept the same to generate predictions. The cAMP signalling profile and the kinetics of receptor species were simulated to show any differences in profile when THC or WIN55,212-2, as opposed to CP55940, is in combination with ORG.

### Data analysis

All empirical data analysis was performed using GraphPad Prism v8. Kinetic traces showing inverse BRET ratios were expressed as mean ± SD. Lag time of cAMP disinhibition by ORG was analysed using a two-way ANOVA assuming sphericity with Šídák’s multiple comparison tests (*p* < 0.05).

## Results and discussion

### Can the model predict new experimental data?

The unified mathematical model published by Yang et al. (2023) was built to describe cAMP inhibition and internalisation of hCB_1_ stimulated with CP55940 in combination with ORG (Cawston et al. 2013). This mathematical model includes the existence of a transitional state of hCB_1_ occupied by CP55940 and ORG, which cannot signal through G proteins but can undergo internalisation (Yang et al. 2023). The idea that hCB_1_ is stabilised into a unique conformation when bound simultaneously by CP55940 and ORG has been proposed throughout the literature (Fay and Farrens 2012, 2015; Shao et al. 2019).

To validate the mechanistic assumptions behind this model, the ability of the model to predict new cAMP inhibition data was tested. The initial data (Cawston et al. 2013) utilised a single concentration of CP55940 (1 μM) and increasing concentrations of ORG. Our initial question was therefore to ask if the model could predict the response to submaximal concentrations of CP55940.

**Fig. 3:**
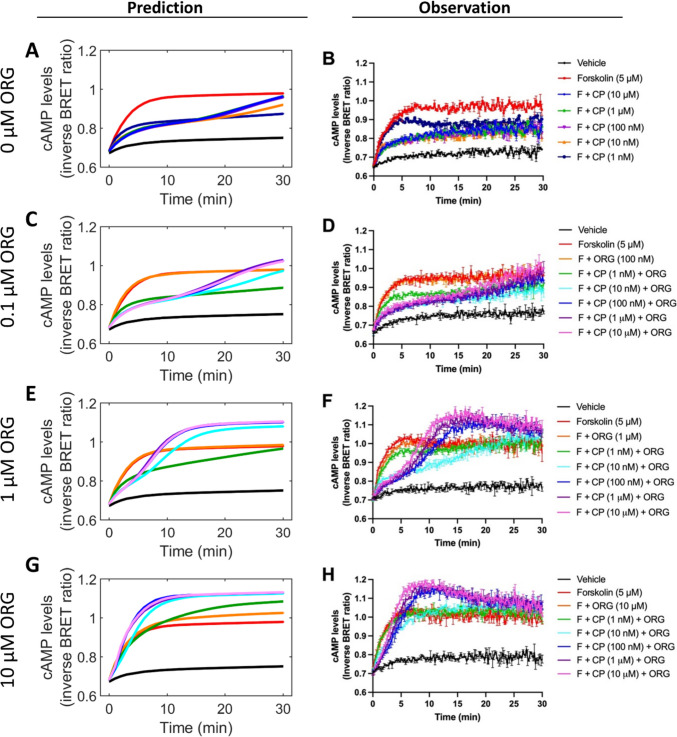
cAMP inhibition by CP55940 in the presence of ORG — comparisons of model predictions and experimental data in hCB_1_-HEK293 cells. Model predictions for cAMP inhibition by varying concentrations of CP55940 (CP) alone (**A**) or in the presence of 100 nM (**C**), 1 µM (**E**), and 10 µM ORG27569 (ORG) (**G**) and associated experimental data (**B**, **D**, **F**, **H**). All conditions are performed in the presence of 5 µM forskolin (**F**). Empirical data is expressed as mean ± SD and is representative data from technical duplicates (*n* = 3)

Firstly, a concentration series of CP55940 in the absence of ORG was investigated and compared to model predictions. A concentration dependent reversal of cAMP inhibition (i.e., disinhibition) by CP55940 was observed in the presence of ORG. The model predicts that in the presence of 100 nM ORG, all concentrations of CP55940 tested produce strong inhibition of cAMP for the first 15 min, before the curves increase toward forskolin, such that curves containing ORG had a kinetic lag in cAMP disinhibition of roughly 15 min (Fig. 3C). This abrogation of the CP55940 effect was dependent on the concentration of CP55940, becoming more pronounced as the CP55940 concentration increased (Fig. 3C). In vitro, a very similar trend was observed; all concentrations of CP55940 in the presence of 100 nM ORG showed complete inhibition of cAMP for the first 15 min, followed by an inhibition of this response occurring after 15 min as the curves increase toward forskolin alone (Fig. 3D). The model simulates a 50% line of prediction surrounded by 95% confidence interval bands from full covariance matrices. All in vitro experimental data of cAMP inhibition by varying concentrations of CP55940 in the presence of 100 nM ORG fit within the 95% confidence interval predicted by the model, therefore validating the mechanistic assumptions of the model (Supplementary Fig. 1).

In the presence of 1 µM ORG, the model predicts that cAMP inhibition by the three highest concentrations of CP55940 (100 nM, 1 µM and 10 µM) is reversed after 5 min (a 5-min kinetic lag in cAMP disinhibition, Fig. 3E). The addition of ORG (1 µM) causes cAMP to increase above the forskolin alone condition at approximately 5 min, with 10 nM CP55940 increasing slightly slower at approximately 7 min (Fig. 3E). In vitro, a similar trend was observed where 1 µM ORG in the presence of all concentrations of CP55940 showed complete inhibition of cAMP for the first 5 min. This complete cAMP inhibition was inhibited by 1 µM ORG in the presence of high concentrations of CP55940 (100 nM, 1 µM and 10 µM) after 5 min, where the curves increase toward forskolin alone and result in inverse agonism by 7–10 min (Fig. 3F). One subtle difference in the model predictions and experimental data was observed with 10 nM CP55940 in combination with 1 µM ORG, where the disinhibition of cAMP inhibition occurred at a slower rate than was predicted by the model. This could be due to the potency of CP55940 being higher in the model than the potency in vitro, and therefore, the extent of inverse agonism produced by 1 µM ORG is also higher in model simulations. In vitro experimental data showing cAMP inhibition by varying concentrations of CP55940 in the presence of 1 µM ORG fits within the 95% confidence interval predicted by the model, therefore validating the mechanistic assumptions of the model (Supplementary Fig. 2).

In the presence of 10 µM ORG, the model predicts that there is no inhibition of cAMP at any concentration of CP55940; instead, inverse agonism is observed (Fig. 3G). The addition of ORG (10 µM) causes cAMP to increase above the forskolin alone condition immediately for all concentrations of CP55940, with lowest concentration of CP55940 (1 nM) taking the longest time to reach the same level of inverse agonism (Fig. 3G). In vitro, a similar trend was observed such that no kinetic lag in cAMP disinhibition was observed with 10 µM ORG in the presence of all concentrations of CP55940 (Fig. 3H). The increase in cAMP was observed to occur at a slower rate than that produced by forskolin alone, and the extent of inverse agonism by ORG in combination with CP55940 was equal or greater than the cAMP stimulation produced by forskolin alone (Fig. 3H). All in vitro experimental data showing cAMP levels by varying concentrations of CP55940 in the presence of 10 µM ORG fit within the 95% confidence interval predicted by the model, therefore validating the mechanistic assumptions of the model (Supplementary Fig. 3).

One qualitative difference observed between model simulations and in vitro experimental data was an initial overshoot of cAMP; however, as this also occurred in forskolin only conditions, this phenomenon was not considered important for the mechanism of action of ORG (Supplementary Figs. 1-3). Furthermore, this phenomenon was not obvious in previous datasets used for building the model (Cawston et al. 2013; Yang et al. 2023). The ability of the model to predict novel cAMP inhibition data of CP55940 in combination with ORG at varying concentrations of CP55940 validates the mechanistic assumptions of the model and proves it has the ability to accurately simulate new experimental data.

### Testing the model-based mechanism for the lag in cAMP disinhibition by ORG

Having validated that the model could be used to simulate novel data, we then aimed to test the mechanism underlying the kinetic lag in cAMP disinhibition by ORG, arising from the model (Yang et al. 2023).

**Fig. 4:**
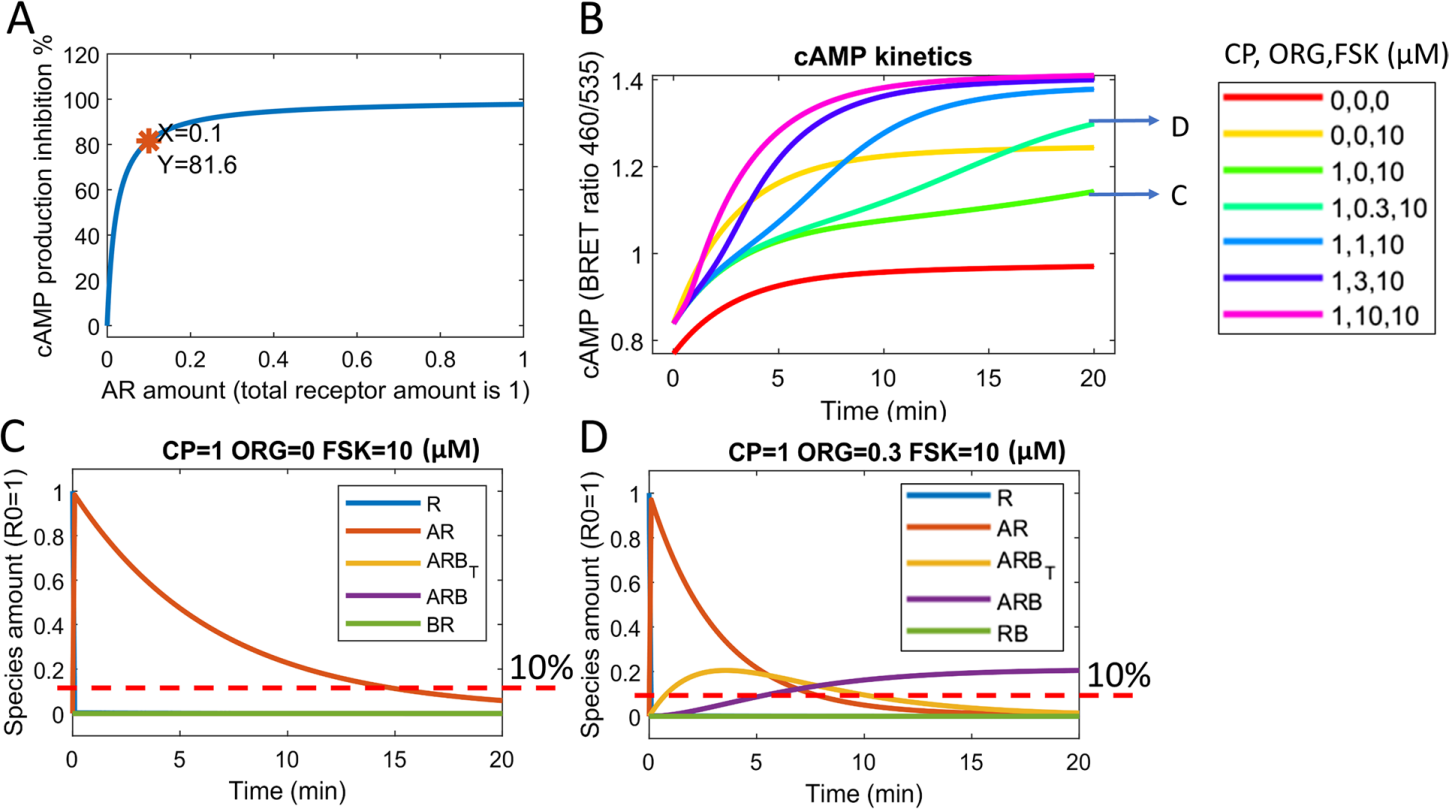
Model simulations investigating receptor species and their relationship to cAMP inhibition. Relationship between the quantity of *AR* and the inhibition of cAMP (**A**), predicted cAMP signalling data of CP55940 alone (green) and in the presence of increasing concentrations of ORG (**B**), predicted receptor species present when CP55940 is added to HEK293-hCB_1_ cells alone (**C**) and in combination with 300 nM ORG (**D**), using kinetic cAMP data published in Cawston et al. (2013)

In the model (Fig. 1), there are two receptor species that contribute to the inhibition of cAMP; agonist bound receptor (*AR*) and constitutively active receptor (*R*) (Yang et al. 2023). The total combined inhibition by these species (*I*) affects cAMP production and can be defined using an E_MAX_ model. We used published cAMP inhibition data to simulate the amount of each receptor species over the time-course of cAMP experiments (20 min) to investigate how each species contributes to total cAMP inhibition (Cawston et al. 2013). When 1 µM CP55940 is added alone, there are two species, *AR* and *R*. There is a fast increase in *AR*, followed by an exponential decrease over time and a rapid decrease in *R* over the first minute (Fig. 4C). This explains the high affinity of the agonist CP55940 and its ability to rapidly occupy unbound receptor. Also, the model-estimated efficacy of  *AR* was much larger than *R*. In the model equation of $I=R+\varepsilon_c\cdot\;AR$, *I* is the total combined inhibition effect and ${\varepsilon }_{c}$ is the intrinsic efficacy of *AR*. The *AR* efficacy ${\varepsilon }_{c}$ equalled to 45.7, while the efficacy of *R* was 1. As *AR* contributes a much larger proportion of total cAMP inhibition than *R* in this case, we can ignore the cAMP inhibition of *R* and simulate the relationship between the quantity of *AR* and the percentage of cAMP inhibition, although cAMP inhibition by *R* is included in all other model calculations (Yang et al. 2023). We found that when CP55940 binds to 10% of avaliable receptors (*AR=0.1)* 82% inhibition of cAMP is observed, meaning that very low receptor occupancy (roughly 20%) is required to cause full inhibition of cAMP (Fig. 4A).

Over the experimental time-course, when CP55940 (1 µM) is added to the system, *AR* stays above 10% for the first 15 min; therefore, full cAMP inhibition would be predicted to be observed over the whole time-course of this assay (Fig. 4C). When 1 µM CP55940 is added in combination with 300 nM ORG, there are three different species present, *ARB*_*T*_, *ARB*, and *RB* (Fig. 4D). For the first 5 min, most receptors are bound only by CP55940 (*AR*), and as the amount of *AR* is far greater than 10% of R_0_, small changes of ORG-bound populations will not affect total inhibition of cAMP. However, after 7 minutes, receptor internalisation and accumulation of *ARB*_*T*_ and *ARB* result in a decrease in *AR* to below 10% of initial total receptor amount R_0_ (Fig. 4D). As there is less *AR* in the population and both *ARB*_*T*_ and *ARB* do not contribute to cAMP inhibition, cAMP inhibition is not saturated and is sensitive to *AR* change (Fig. 4A). Therefore, small changes in ORG-bound species (which will further reduce *AR*) will affect total cAMP inhibition, and therefore, we hypothesised the lag in cAMP disinhibition will no longer be present at this time point.

**Fig. 5:**
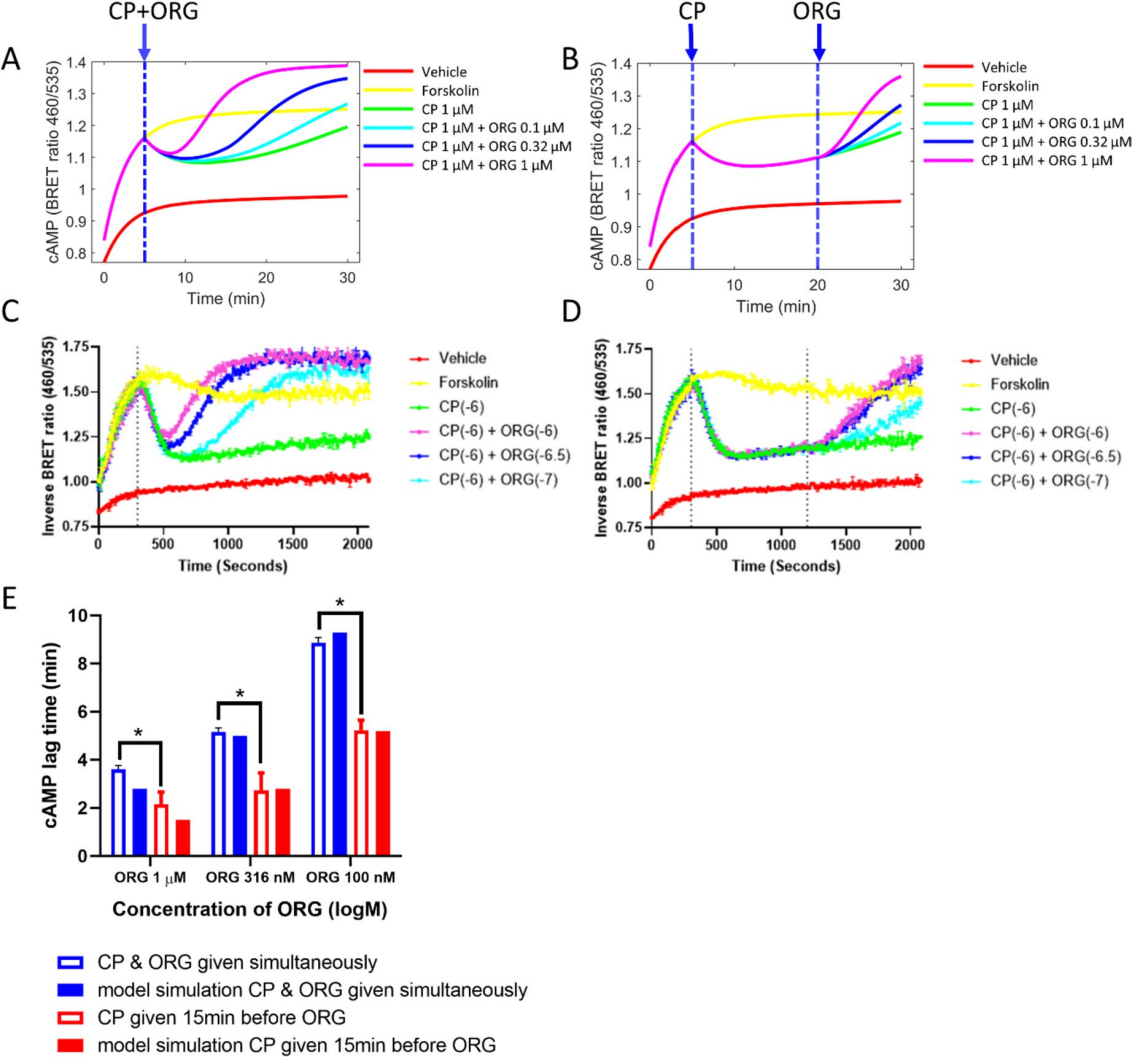
Decreasing the kinetic lag in cAMP disinhibition by ORG in combination with CP55940. Model predictions of cAMP inhibition in HEK293-hCB_1_ cells when CP55940 and ORG are added simultaneously (**A**) and when CP55940 is added 15 min prior to the addition of ORG (**B**), compared to empirical data (**C**, **D** respectively**)**. Empirical data is expressed as mean ± SD and is representative data from technical duplicates (*n* = 3). Kinetic lag times were predicted using the model and compared to calculated lag times from empirical data expressed as mean ± SEM (*n* = 3;** E**). Statistical analysis (comparing lag time when CP55940 and ORG were given simultaneously, and when CP55940 was given 15 min prior to ORG) was performed in GraphPad Prism using a repeated measures two-way ANOVA with Šidák’s post-hoc test; *p* values indicated as * < 0.05

Due to the above analysis, we infer that when *AR* is less than 10% R_0_, cAMP inhibition is sensitive to changes in ORG-bound receptor populations. If this is true, when ORG is added to the system, there will be a shorter kinetic lag in cAMP disinhibtion at lower receptor level. To test this hypothesis, we designed an in vitro experiment that used CP55940-induced hCB_1_ internalisation to decrease *AR* (Zhu et al. 2019). CP55940 (1 µM) was added to HEK293-hCB_1_ cells and incubated for 15 min to decrease *AR* to roughly 10% (Fig. 5B), prior to the addition of ORG (Fig. 5D). This was then compared to a control experiment where CP55940 and ORG were added to HEK293-hCB_1_ cells simultaneously (Fig. 5C). Using the model, we simulated cAMP inhibition data and produced lag times for each combination of CP55940 and ORG (Fig. 5A, B). The in vitro experimental data very closely matched the model simulated curves of cAMP inhibition, thereby supporting our hypothesis that the kinetic lag in cAMP disinhibition would be reduced following a reduction in receptor number (Fig. 5). The model predicted that when CP55940 and ORG (1 µM, 316 nM, and 100 nM) are added simultaneously, the lag in cAMP disinhibition is greater than when CP55940 is added 15 min prior to the addition of ORG. This was tested experimentally in vitro for three different ORG concentrations (Fig. 5), and lag times were quantified (Fig. 5E). For all concentrations of ORG (1 µM, 316 nM, and 100 nM), the cAMP lag time was statistically greater when CP55940 and ORG were added simultaneously, compared to when CP55940 was added 15 min prior to ORG (Fig. 5). This finding was consistent with the model predictions (Fig. 5), and lag times calculated using in vitro experimental results were very similar to that predicted by the model, further validating our hypothesis (Fig. 5). Using the mathematical model in conjunction with an experimental approach provides an exciting opportunity to probe mechanism of action in a unique way, such as exploring the relationship between receptor conformational species, that cannot be observed in the real world through in vitro experimentation.

### Investigating probe dependence of ORG

Allosteric modulators are thought to have unique relationships with different orthosteric agonists, a concept known as probe dependence (Wootten et al. 2013). ORG has been suggested to be a less potent inhibitor of WIN55,212-2-induced cAMP inhibition compared to CP55940 (Baillie et al. 2013). However, it is challenging to identify whether the differing potencies of ORG in combination with different orthosteric agonists are determined predominately by the efficacy and affinity of the orthosteric ligand, or whether it is due to ORG having distinct interactions with hCB_1_ depending on the orthosteric ligand present. Unique conformational states and the characteristics of hCB_1_ bound simultaneously by ORG and an orthosteric ligand are challenging to quantify in vitro; therefore, the mechanism of probe dependence remains unknown. We investigated whether this model can be used to predict the relationship between ORG and other orthosteric agonists, to gain further insight into the mechanisms behind the proposed probe dependence with ORG.

**Fig. 6:**
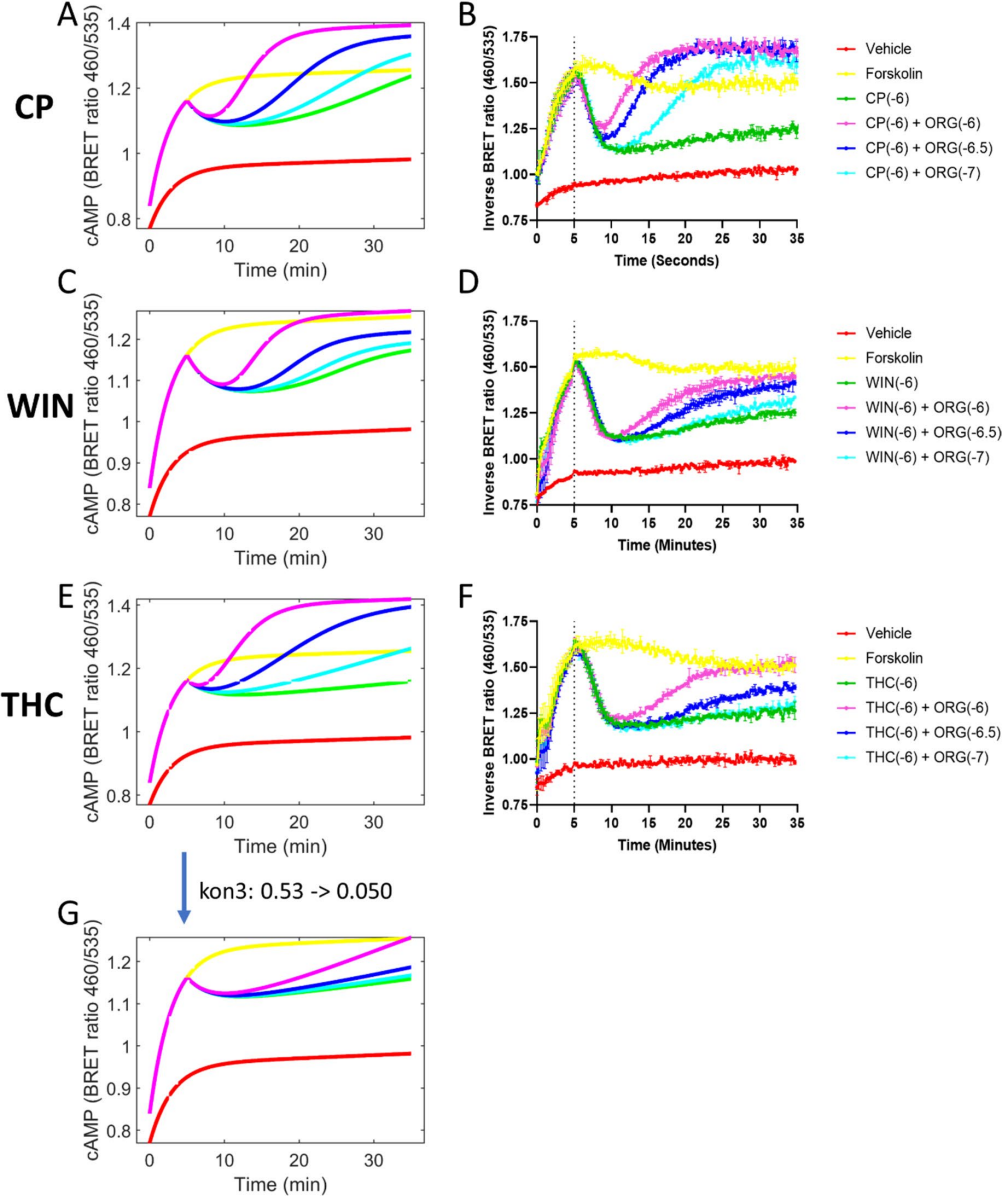
cAMP inhibition by different orthosteric agonists in combination with ORG. Model predictions of cAMP inhibition by CP55940 (**A**), WIN55,212-2 (**C**), and THC (**E**), compared to empirical cAMP data in HEK293-hCB_1_ cells (**B**, **D**, **F**, respectively). Model predictions of THC in combination with ORG after decreasing k_on3_ to represent ORG binding to THC-hCB_1_ with decreased affinity compared to CP55940-hCB_1_ (**G**). Empirical data is expressed as mean ± SD and is representative data from three independent replicates performed with technical duplicates (*n* = 3)

We chose to explore ORG in combination with the hCB_1_ high efficacy agonist WIN55,212-2, and the partial agonist THC, as these compounds along with CP55940 cover a broad range of potencies and efficacies of cannabinoid agonists (Finlay et al. 2019). Three orthosteric agonist specific parameters were changed according to published data for THC and WIN55,212-2, and all other model parameters were unchanged to simulate cAMP inhibition predictions (Table 1; Fig. 1; Fig. 6A, C, E). If the model accurately simulates cAMP inhibition data for ORG in combination with WIN55,212-2 and THC given parameters specific to the orthosteric agonist (affinity/efficacy), this would indicate that ORG has a similar relationship with CP55940 and WIN55,212-2 or THC (i.e. no probe dependence). If not, this would be an example of probe dependence, as the interaction between the orthosteric agonist and ORG cannot accurately be predicted through estimation of parameters exclusive to the orthosteric agonist, indicating a unique interaction of ORG with different orthosteric agonists. Interaction parameters (those that relate to both ORG and the orthosteric agonist) can then be modified to explore this further. If changing both agonist and interaction parameters did not accurately simulate data this would indicate that fundamental changes in the model structure are required and question its broader utility.

**Table 1:** Parameters modified for model simulation of multiple orthosteric ligands

	Orthosteric parameters			Interaction parameters
Agonist	$pKd$(−logM)	${k}_{int}$(min^−1^)	${\text{log}}10({\varepsilon }_{c})$	${k}_{on3}$
CP	8.6 (estimated)	0.15 (estimated)	1.66^3^	0.53 (estimated)
WIN	7.7^1^	0.28^3^	2.63^3^	0.53^4^
THC	8.0^2^	0.029^3^	0.85^3^	0.53^4^ OR 0.05^5^

References for the parameters:

^1^(Brizzi et al. 2007)

^2^(Cheng et al. 2012)

^3^(Zhu et al. 2020)

^4^Parameter was fixed as the same value of CP + ORG

^5^Parameter was calibrated by aligning experimental data

The model was first used to simulate cAMP inhibition data of WIN55,212-2 in combination with ORG, where it was observed that WIN55,212-2 in combination with ORG results in an increased lag time and decreased extent of inverse agonism compared to CP55940 in combination with ORG (Fig. 6A, C). Empirical data of cAMP inhibition by WIN55,212-2 in combination with ORG were found to match well with the model simulations, such that the calculated lag times are slightly longer and the extent of inverse agonism (cAMP levels higher than forskolin 10 µM) was significantly less than that observed with CP55940 in combination with ORG (Fig. 6D). The differences in inverse agonism align with the cAMP inhibition finding published in Baillie et al. (2013), where 100 nM ORG in combination with CP55940 produced cAMP levels greater than forskolin; however, no concentration of ORG (up to 1 µM) in combination with WIN55,212-2 resulted in cAMP levels greater than forskolin. From the model perspective, the ability to fit data for WIN55,212-2 in combination with ORG when only orthosteric parameters were changed is evidence that the differences in lag time and inverse agonism can be explained by agonist efficacy. WIN55,212-2 has higher efficacy than CP55940 (it has an epsilon, “intrinsic efficacy”, 10-fold higher); therefore, a smaller proportion of receptors are required to be occupied to produce maximal cAMP inhibition. This means that *AR* needs to be decreased to a greater extent in the presence of WIN55,212-2 compared to CP55940 to observe the effect of ORG, and therefore, the kinetic lag in cAMP disinhibition is increased. Due to the high efficacy of WIN55,212-2, very low levels of *AR* will still generate stimulus resulting in the inhibition of cAMP. To observe inverse agonism, levels of *AR* would need to be depleted down to an unachievable level due to receptor synthesis and internalisation; therefore, in the current system, inverse agonism is not observed with WIN55,212-2 in combination with ORG.

As the in vitro experimental results closely match the model simulations of WIN55,212-2 in combination with ORG, it can be concluded that ORG has a similar relationship with both WIN55,212-2 and CP55940.

Changing only agonist specific parameters for THC, however, did not result in a good prediction of the response to THC in combination with ORG (Fig. 6E). Due to the low efficacy of THC, the model predicted that in combination with ORG, the shortest lag times and highest level of inverse agonism would be observed (Fig. 6E). When the results were tested experimentally, surprisingly, THC in combination with ORG resulted in longer lag times and no inverse agonism when compared to CP55940 in combination with ORG (Fig. 6F). We hypothesised that this might reflect ORG having a different affinity for hCB_1_ when bound to either THC or CP55940. The potency of ORG has been found to be increased in the presence of agonist, indicating that ORG has higher affinity for the active conformation of hCB_1_ (Cawston et al. 2013). Partial agonists cause a lower proportion of receptors in the population to be stabilised into the active state and/or cause a less active conformation of hCB_1_ to be stabilised upon binding. As ORG has greater affinity for the active conformation of hCB_1_, we hypothesised that ORG would have decreased affinity for THC-hCB_1_ compared to CP55940-hCB_1_, on the individual receptor or total receptor population level. In the model, the affinity of ORG for THC-bound hCB_1_ is defined as k_on3_ (the rate of the allosteric ligand binding to the agonist occupied receptor; Yang et al. 2023).

To investigate whether ORG has decreased affinity for THC-hCB_1_ and to evaluate whether the change in affinity was sufficient to accurately model ORG in combination with THC, k_on3_ was decreased ten-fold to 0.05 µM^−1^∙min^−1^ (for CP55940, original k_on3_ = 0.528 µM^−1^∙min^−1^). The model predictions following this change were consistent to experimental results observed in vitro, such that the cAMP lag is increased compared to CP55940 in combination with ORG, and no inverse agonism is observed (Fig. 6G). This indicates potential probe dependence. As there were no fundamental changes in model structure required for the model to accurately predict cAMP inhibition data for ORG in combination with different orthosteric ligands, we hypothesise that the mechanism of the interaction is consistent between agonists, with efficacy differences sufficient to explain varied cAMP inhibition results with different orthosteric agonists in combination with ORG.

The exact mechanism by which ligands generate partial agonism at hCB_1_ is not fully defined. Partial agonists may stabilise less receptor in the active state, or they may stabilise a unique state. Recently, it has been proposed that partial agonists of hCB_1_, such as anandamide, stabilise a unique active conformation compared to high efficacy synthetic agonists, such as AMB-FUBINACA (Krishna Kumar et al. 2023). Specifically, anandamide was found to stabilise the twin-toggle switch residue W356^6.48^ into the active conformation to a significantly lower extent than AMB-FUBINACA, while also having decreased interactions with TMH2 compared to AMB-FUBINCA (Krishna Kumar et al. 2023). This indicates that ligands of different efficacies can stabilise unique conformations of hCB_1_, and therefore, it is possible that ORG will have differing affinity to these unique conformations. This could have implications for how ORG modulates endocannabinoid action in the brain. There are two endogenous orthosteric agonists at hCB_1_, the partial agonist anandamide, and the high efficacy agonist 2-arachidonylglycerol (2-AG; Katona et al. 2008). Thus, ORG may have lower affinity for AEA-hCB_1_ compared to 2-AG-hCB_1_. However, a limitation of investigating probe dependence in this way is that the changes in parameter values are done via parameter calibration using experimental datasets for cAMP inhibition only. For more accurate modelling, parameter estimation could be performed if complete datasets of cAMP inhibition, radioligand binding, and internalisation of CB_1_ were used for different orthosteric agonists in combination with ORG.

## Conclusions

ORG is an allosteric modulator with a complex mechanism of action, characterised to result in a unique kinetic lag in cAMP disinhibition (Price et al. 2005; Cawston et al. 2013). We have used a unified mathematical model to aid in the investigation of the mechanism of action of ORG at hCB_1_. By using the model to investigate the interaction between different receptor species and cAMP inhibition, we determined the mechanism behind the kinetic lag in cAMP disinhibition by ORG is likely due to receptor reserve in the cAMP pathway. By decreasing total receptor number cAMP is more sensitive to changes in ORG-bound receptor species, and the kinetic lag is decreased (Fig. 5E). The model was successfully expanded to be used in the investigation of multiple orthosteric agonists by testing and iteratively changing agonist specific parameters. The different profiles of cAMP inhibition by orthosteric agonists in combination with ORG were found to be controlled predominately by orthosteric agonist efficacy; however, probe dependence was observed with ORG in combination with THC. Therefore, this predictive modelling strategy is important, as allosteric modulators can cause different responses depending on the orthosteric ligand, and this work suggests that this is due to differing affinity of the allosteric modulator for the receptor when bound to a partial agonist. Expanding the model beyond CP55940 and ORG significantly increases the application potential of the model and allows for further adaptations to be investigated in the future, such as expanding the model to include other allosteric modulators. This model can facilitate scientific research due to the ability to predict the results of in vitro experiments and therefore aid in experimental design and evaluation of ligand mechanism of action.

### Supplementary Information

Below is the link to the electronic supplementary material.

Supplementary file1 (DOCX 3515 KB)

## Data Availability

Data supporting the findings of this study are available from the corresponding author upon reasonable request.
