# Supplementary material for: Insight into the mechanism of action of ORG27569 at the cannabinoid type one receptor utilising a unified mathematical model
Source: Naunyn Schmiedebergs Arch Pharmacol. 2024 Jan 16;397(7):5105–18. doi: 10.1007/s00210-023-02923-6 (PMC11166842; doi:10.1007/s00210-023-02923-6)
Supplement: Supplementary file 1 — Supplementary file1 (DOCX 3515 KB) [file 210_2023_2923_MOESM1_ESM.docx]

**Supplementary information:**

**Title:** Insight into the mechanism of action of ORG27569 at the cannabinoid type one receptor utilising a unified mathematical model.

**Running Title:** Insight into the mechanism of action of ORG27569 at the cannabinoid type one receptor utilising a unified mathematical model.

**Authors:** Hayley M. Green^a^, Liang Yang^b^, Xiao Zhu^c^, David B. Finlay^a^, Stephen B. Duffull^b,d^, Michelle Glass^a^*

^a^ Department of Pharmacology and Toxicology, School of Biomedical Sciences, University of Otago, Dunedin, New Zealand

^b^ Otago Pharmacometrics Group, School of Pharmacy, University of Otago, Dunedin, New Zealand

^c^ Department of Clinical Pharmacy and Pharmacy Administration, School of Pharmacy, Fudan University, Shanghai, China

^d^ Princeton, NJ, USA

***Corresponding author:**

Professor Michelle Glass

Department of Pharmacology and Toxicology, University of Otago, PO Box 56, Dunedin, New Zealand 9054

+64 3 479 8524; [michelle.glass@otago.ac.nz](mailto:michelle.glass@otago.ac.nz)

**Figure S1: Validation of the model to simulate novel cAMP data of CP55940 and 100 nM ORG27569 at the cannabinoid type 1 receptor.** Global sensitivity analysis compared to empirical data, such that 95% confidence intervals generated by full covariance metrics are shown as the shaded bands, and experimental data is shown by the thick line (A). Experimental results in (A) are combined in (B), and the corresponding model predictions are combined in (C).


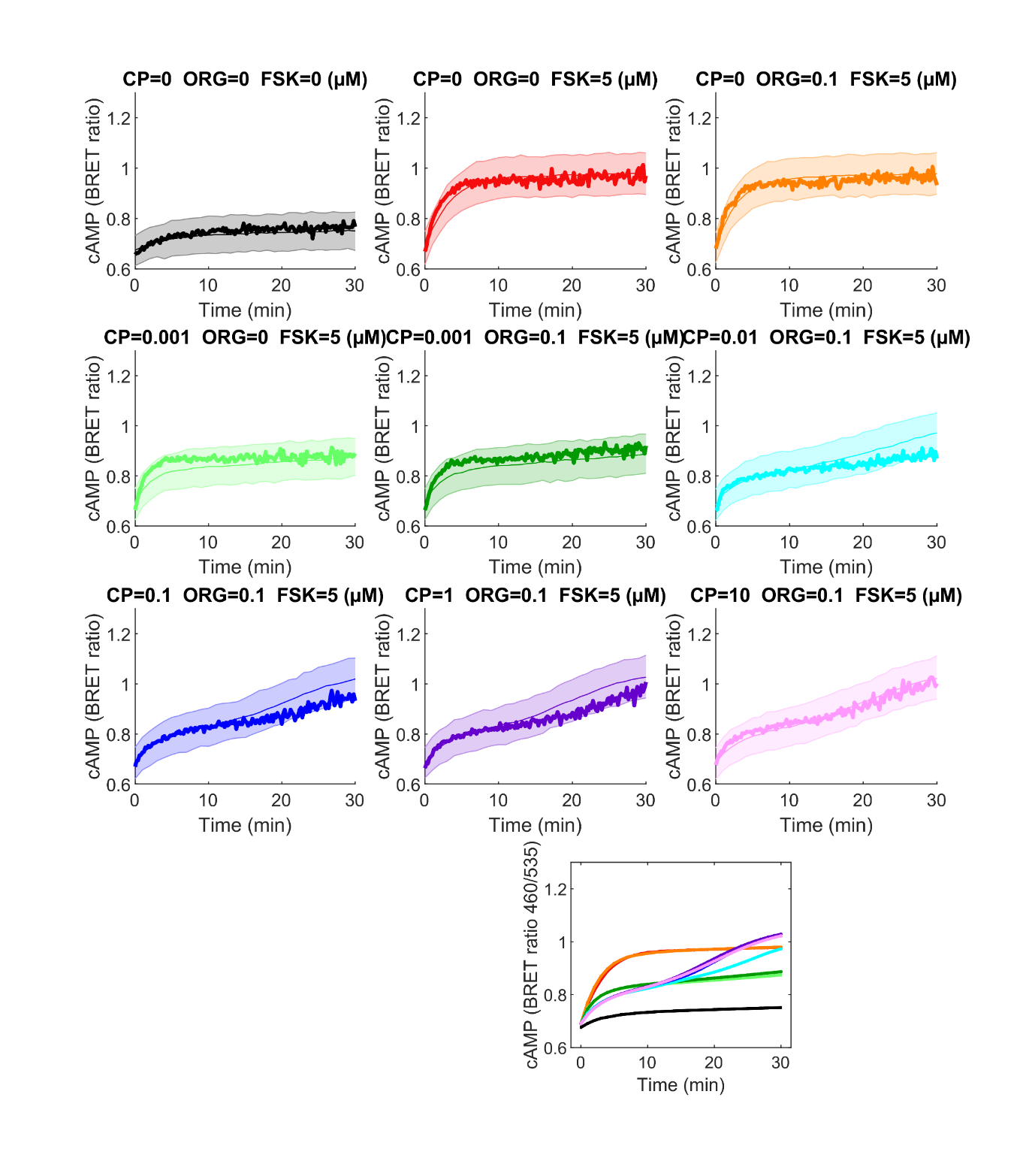

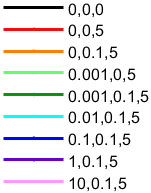


CP, ORG, FSK (µM)

**Observation**

**Prediction**

A

C

B


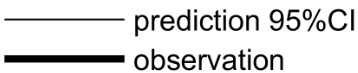


**Figure S2: Validation of the model to simulate novel cAMP data of CP55940 and 1 µM ORG27569 at the cannabinoid type 1 receptor.** Global sensitivity analysis compared to empirical data, such that 95% confidence intervals generated by full covariance metrics are shown as the shaded bands, and experimental data is shown by the thick line (A). Experimental results in (A) are combined in (B), and the corresponding model predictions are combined in (C).


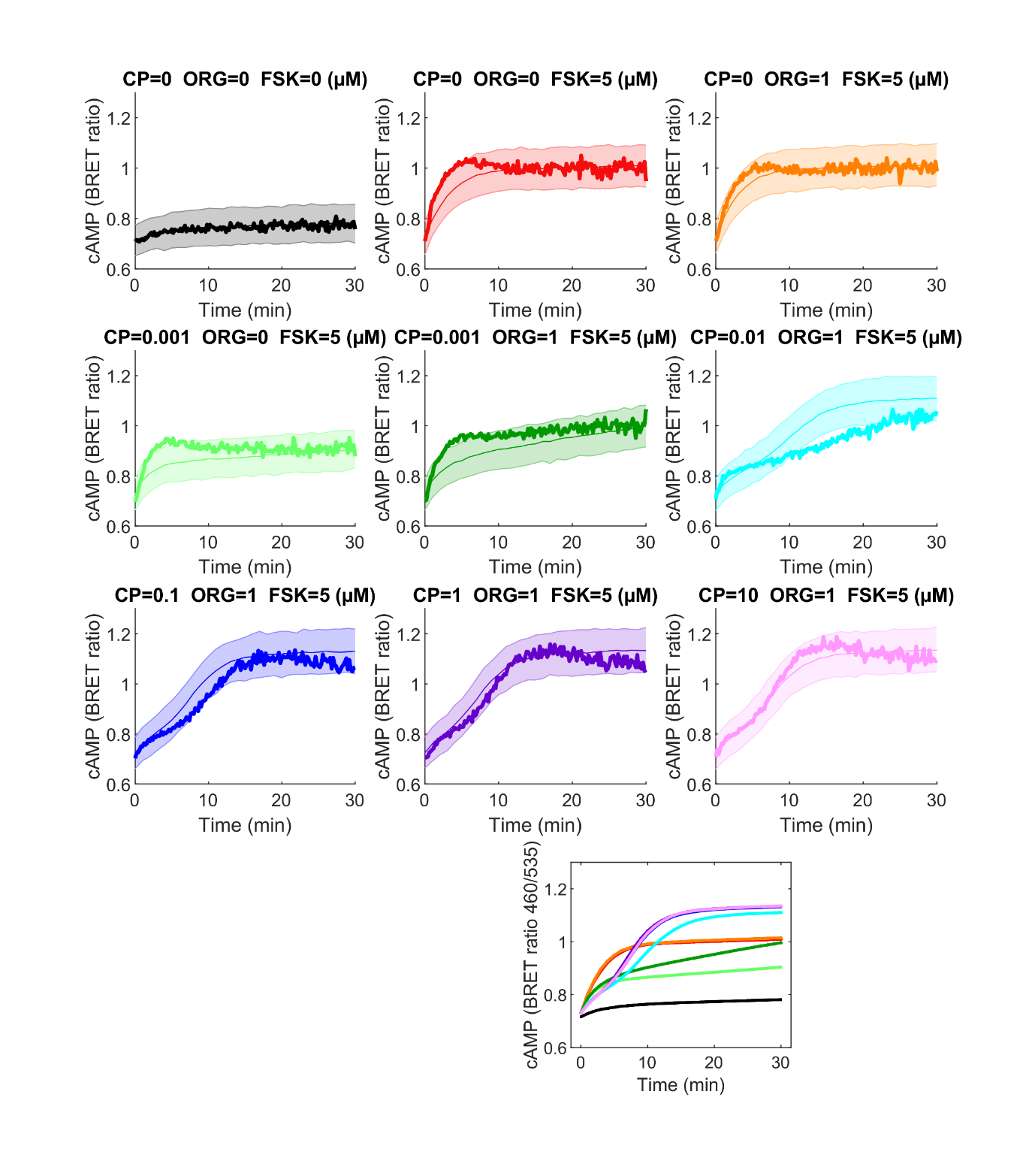


CP, ORG, FSK (µM)

**Observation**

**Prediction**


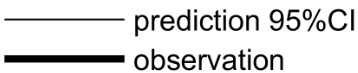

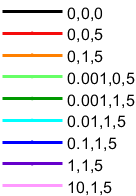


A

C

B

**Figure S3: Validation of the model to simulate novel cAMP data of CP55940 and 10 µM ORG27569 at the cannabinoid type 1 receptor.** Global sensitivity analysis compared to empirical data, such that 95% confidence intervals generated by full covariance metrics are shown as the shaded bands, and experimental data is shown by the thick line (A). Experimental results in (A) are combined in (B), and the corresponding model predictions are combined in (C).
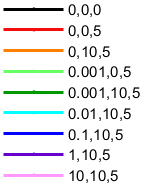

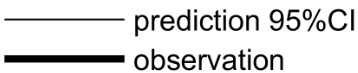

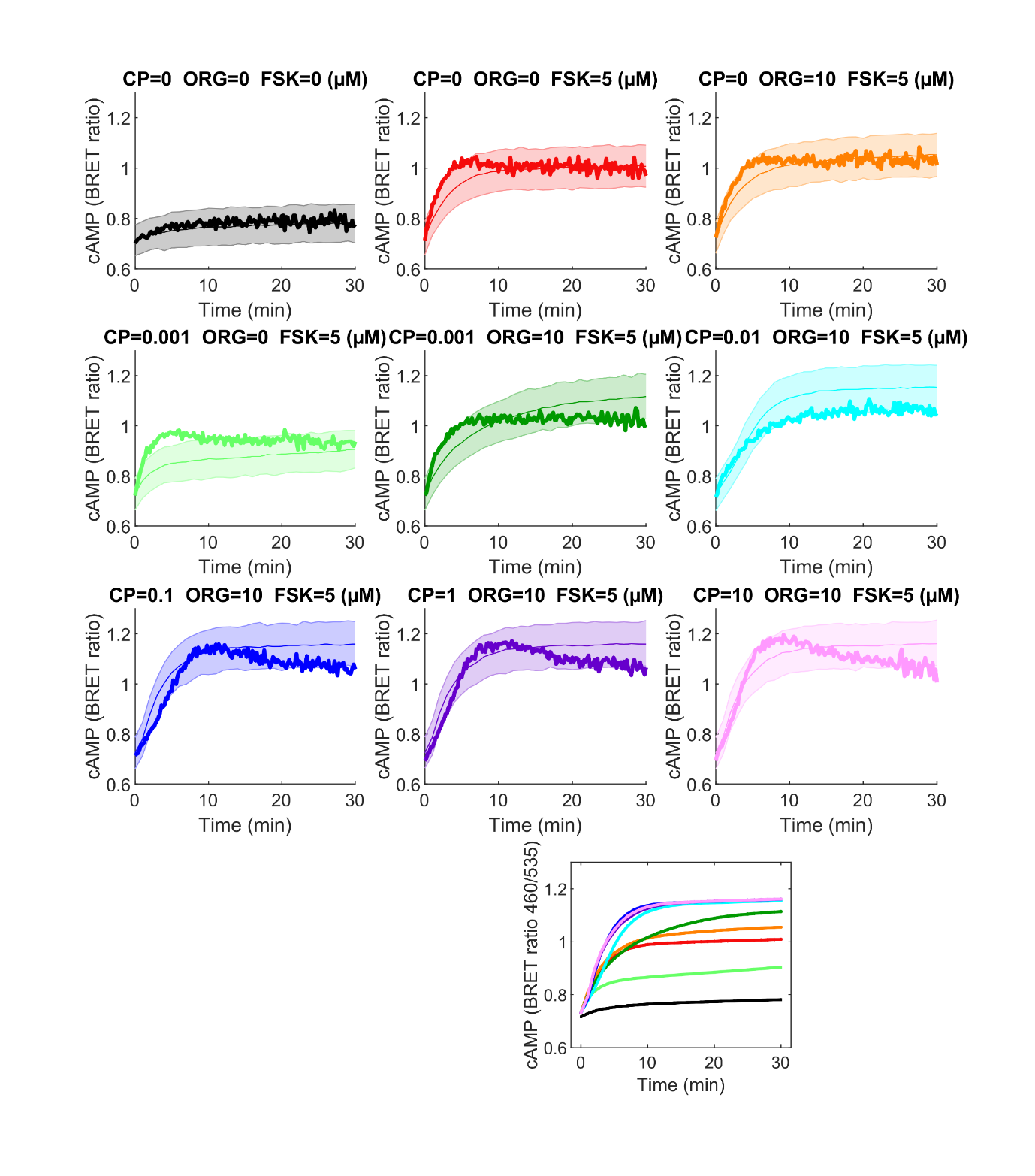


**Prediction**

**Observation**

CP, ORG, FSK (µM)

A

B

C
